# Animal-Assisted Therapy in Middle-Aged and Older Patients With Schizophrenia: A Randomized Controlled Trial

**DOI:** 10.3389/fpsyt.2021.713623

**Published:** 2021-08-03

**Authors:** Tzu-Ting Chen, Ton-Lin Hsieh, Mei-Li Chen, Wei-Ting Tseng, Chi-Fa Hung, Chyi-Rong Chen

**Affiliations:** ^1^Department of Psychiatry, Kaohsiung Chang Gung Memorial Hospital and Chang Gung University College of Medicine, Kaohsiung, Taiwan; ^2^School of Occupational Therapy, College of Medicine, National Taiwan University, Taipei, Taiwan; ^3^Professional Animal-Assisted Therapy Association of Taiwan, Taipei, Taiwan; ^4^Department of Nursing, School of Nursing, National Taipei University of Nursing and Health Sciences, Taipei, Taiwan

**Keywords:** animal assisted therapy (AAT), aging, schizophrenia, negative symptom, Adjunct therapy

## Abstract

**Objective:** Animal-assisted therapy (AAT) has the potential to improve the symptomology, negative emotions, and level of well-being in older adults, as well as patients with mental illness. However, there remains limited evidence supporting the treatment efficacy of AAT in middle-aged and older adults with schizophrenia. Therefore, this study implemented a randomized controlled trial to assess the efficacy of a 12-week AAT psychological intervention with dogs for middle-aged and older patients with chronic schizophrenia in a clinical setting.

**Method:** Patients, age ≥ 40 years, with chronic schizophrenia were allocated randomly to either the AAT group or control group. Patients in the AAT group received an additional hour -long AAT session every week for 12 weeks. Patients in the control group received the usual treatment plus an hour long non-animal related intervention. All patients were assessed based on primary outcome measures before and after the 12-week intervention, including the Positive and Negative Syndrome Scale (PANSS), Depression Anxiety Stress Scales Assessment (DASS), and Chinese Happiness Inventory (CHI).

**Results:** Patients who received AAT had greater improvements in the PANSS and DASS-stress subscale scores than the control group (*p* < 0.05). The effect was small (success ratio different, SRD = 0.25) for the PANSS and the DASS-stress subscale (SRD = 0.15). There were no significant differences in the change scores of the CHI between the AAT and control groups (*p* = 0.461).

**Conclusions:** AAT seemed to be effective in reducing psychiatric symptoms and stress levels of middle-aged and older patients with schizophrenia. AAT could be considered as a useful adjunctive therapy to the usual treatment programs.

## Introduction

Schizophrenia is a mental disability characterized by positive symptoms (e.g., delusions and hallucinations), negative symptoms (e.g., apathy and anhedonia) (1), and general psychopathology (e.g., depression and anxiety) (2–5). Schizophrenia has adverse impacts on major areas of life, such as work and interpersonal relations (1). Approximately 1% of population are affected by schizophrenia (6), of which 25% or more will soon be middle-aged and older individuals (7, 8). Schizophrenia is particularly challenging for this age group because these individuals tend to have more severe psychotic symptoms and poorer psychosocial function (9). About 60% of patients with schizophrenia living in the hospital are middle-aged and older (10), dying 10–15 years earlier than the general population (11). Their negative symptoms and cognitive impairment are significantly more severe than those in younger patients (12) and pose a significant challenge to psychiatric treatment (7).

Treatments for schizophrenia typically involve antipsychotic drugs and psychotherapy (13). However, the effectiveness of these treatment is questionable (14–17) because negative and cognitive symptoms often remain problematic (11, 18). Therefore, it is important to seek alternative psychosocial treatments to improve the psychotic symptoms of patients with schizophrenia.

Animal-Assisted Therapy (AAT) has recently garnered increased attention and been used as an adjunct to typical treatments and interventions for patients with mental illness, including schizophrenia (19, 20). AAT is a structured, planned, and goal-oriented therapeutic intervention involving interactions between a patient and an animal (typically a dog), along with a therapist and an animal handler (21, 22). Example AAT activities include taking care of a dog and playing with a dog (19, 20, 23–25). AAT is typically used to improve the symptoms, functioning (emotional, social, and cognitive), and quality of life for patients with mental illness (19, 20, 23, 26–30), In addition, it may be particularly helpful in the treatment of schizophrenia (22). Interacting with animals can also increase oxytocin levels, which has been shown to improve psychiatric symptoms (31, 32). Relaxing human–animal relations may help dampen negative emotions (20, 29). Therapy dogs can serve as emotional mediators to provide support and company (20, 33), and may increase patient quality of life and well-being (29).

Previous studies have used AAT to improve psychiatric symptoms, emotion, and quality of life in patient with schizophrenia, but there still remains insufficient significant evidence demonstrating its effectiveness. Some studies have revealed positive results, with improvements in positive symptoms, negative symptoms, and general psychopathology symptoms (including stress, anxiety, and depression) (20, 23, 25, 29, 34). However, other studies found no significant improvements in motivation (25), general psychopathology symptoms (23), and quality of life (20). Moreover, there were several methodological limitations in previous studies, including the lack of structural AAT programs, small sample sizes, lack of control groups, short durations of intervention, and limited professionals and animals involved in AAT sessions (20, 21). Few studies have targeted middle-aged and older patients with schizophrenia (21). Therefore, the evidence regarding the effectiveness of AAT in middle-aged and older patients with schizophrenia remains inconclusive and insufficient (20, 21). As such, this study sought to evaluate the effects of AAT for middle-aged and older schizophrenia patients on psychotic symptoms, negative emotions, and well-being.

## Method

### Participants

Participants were recruited from a psychiatric rehabilitation ward and the day-care ward of a medical center in Taiwan. Participants met the following criteria: (1) diagnosis of schizophrenia according to the fifth edition of Diagnostic and Statistical Manual of Mental Disorders, (2) age**≥**40 years, and (3) stable physical and psychological health conditions based on clinician assessment. Participants with the following criteria were excluded: (1) severe cognitive impairment (e.g., aphasia or inability to follow three-step directions), (2) animal allergies, (3) history of asthma, (4) coagulation disorders, (5) presenting symptoms of dog-related specific phobia, anxiety disorder, and obsessive-compulsive disorder, and (6) had participated in other clinical trials in the past 6 months.

### Design

**Figure 1:**
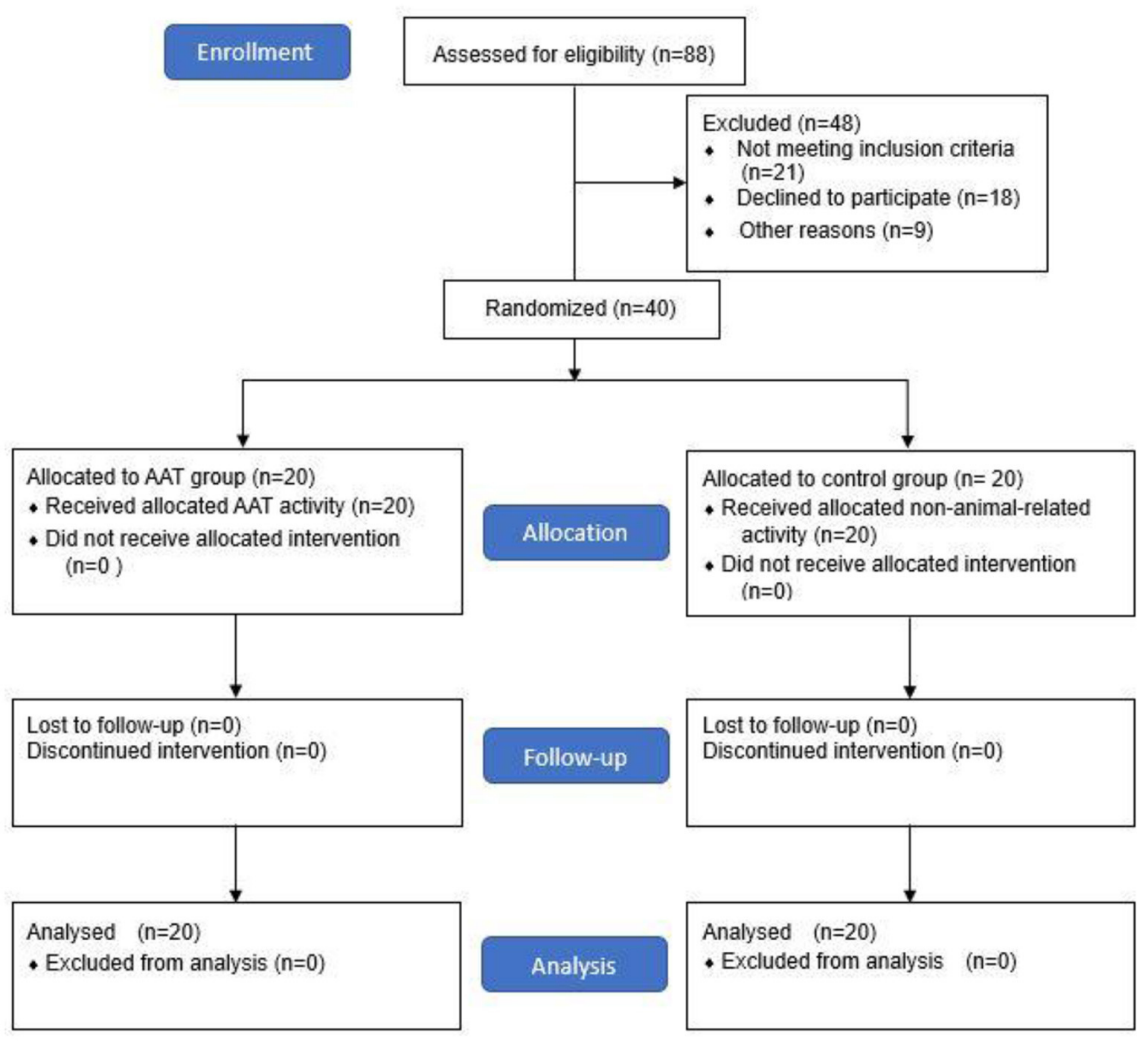
Flow Diagram.

This study implemented a randomized controlled trial (RCT) with parallel-group design and pre-post measurements. Because of the different functional characteristics of patients in day-care and rehabilitation wards, stratification was performed in order to control for confounding variables. Forty patients who met the inclusion criteria were recruited and randomly assigned to the AAT group (intervention group) and control group, with 20 participants in each group (Figure 1). To ensure allocation concealment, participants were randomized by an external clinic using sequentially numbered, opaque sealed envelopes. Stratified randomization was carried out with an online randomizer (www.randomiser.com). Previous studies suggested that group size was best kept small for AAT sessions to ensure quality and safety of treatment; accordingly, participants in the AAT group were further divided into two small groups (groups A and B) to attend the AAT session at the same time. There were no differences in treatment between groups A and B.

### Procedure

Participants in both groups received their usual treatment programs, consisting of nursing interventions, pharmacotherapy, occupational therapy, psychotherapy, sociotherapy, and recreational activities. The AAT group received an additional hour of AAT session for 12 weeks. The control group received a non-animal-related nursing intervention and occupational therapy session of usual treatment programs instead. All participants were assessed 1 week prior to and after the 12-week program by one psychiatric physician and one occupational therapist.

### Measures

#### Positive and Negative Syndrome Scale (PANSS)

The PANSS is widely used to assess the symptom severity of patients with schizophrenia in clinical trials (2–4). The scale comprises 30 items across 3 subscales: the positive subscale (seven items), negative subscale (seven items), and general psychopathology subscale (16 items). Each item is scored on a 7-point scale based on the level of psychopathology present, from 1 (absent) to 7 (extreme). The total score ranges from 30 to 210, with higher scores indicating greater overall symptom severity (35). The validity and reliability of the Chinese Mandarin version of the PANSS has been established in a previous study (36). A minimum total change score of 10.4 was accepted as having responded to treatment (37). The minimum change score for each subscale was assessed based on the total score percentage of each subscale (positive: 2.4, negative: 2.4, general psychopathology: 5.6).

#### Chinese Happiness Inventory (CHI)

The 20-item version of the CHI is a self-report measure of subjective wellbeing for those following the Chinese culture (38, 39). The CHI comprises items with relation to achievement at work, downward social comparisons, peace of mind, optimism, social commitment, positive affect, sense of control, physical fitness, and satisfaction with self in the Chinese society (39, 40). Each item is scored on a 4-point scale from 1 to 4, corresponding to a different level of happiness (37). The total score ranges from 20 to 80, with higher scores indicating greater happiness (37). The CHI has high reliability (41).

#### Depression, Anxiety Stress Scales-21 (DASS-21)

The DASS-21 is a self-report questionnaire used to assess symptoms of depression, anxiety, and stress (42). The DASS-21 comprises 21 items, each describing a negative emotional symptom the participant experienced in the past week (42). Each item is scored on a 4-point scale from 0 (never) to 3 (almost always). Scores of depression, anxiety, and stress are calculated by summing the relevant items. Higher scores indicate more severe negative emotional symptoms. The DASS-21 is a well-established instrument with sufficient reliability and validity (42). The minimal detectable change of total score and each subscale of anxiety, depression, and stress were 7.8, 2.4, 2.2, and 3.2 respectively (43).

### Interventions

#### Personnel

Each AAT session was conducted by an animal-assisted therapist, an occupational therapist, and a dog-handler pair (breeder). Prior to the commencement of the study, the animal-assisted therapist and occupational therapist cooperated to design the intervention protocol to match the needs of the patients. The AAT sessions were primarily led by the animal-assisted therapist, with the help of the occupational therapist as the co-leader, who helped encourage patients to participate. The breeder's job was to instruct their therapy dog to follow the directions of the therapist. All members were sufficiently qualified to effectively carry out the session.

The animal-assisted therapists, therapy dogs, and breeders have been certified by the Professional Animal-Assisted Therapy Association of Taiwan; and the therapy dogs, including Corgi, Labrador Retriever, Maltese, and Shiba Inu, passed the therapy dog test to ensures that they could remain calm in difficult, distracting, and stressful situations. The occupational therapists specialized in psychiatric rehabilitation and were familiar with the global functions of the patients.

#### Animal-Assisted Therapy

The primary goal of the AAT was to improve the negative symptoms (blunted affect, emotional withdrawal, social withdrawal, lack of spontaneity, and flow of conversation) and general psychopathology symptoms (anxiety, depression, uncooperativeness, disorientation, and poor attention) of patients. Secondary goals were aimed at improving positive symptoms (conceptual disorganization, suspiciousness, persecution, and hostility) and patient well-being.

The AAT sessions were conduct in a spacious and quiet classroom, with the participants seated in a semicircle. The animal assisted therapist, occupational therapist, and therapy dog were positioned in front of the participants. The dog approached the participants in turn, and each participant walked the dog around the classroom.

Each AAT session was carried out according to a similar overall structure: 15-min warm-up, 45-min therapeutic activities, and 5-min feedback. In the warm-up, the animal assisted therapist started by greeting each participant, introduced the therapy dog, reviewed the contents of the last session, and oriented participants to the therapeutic activities.

There were four types of therapeutic activities carried out to achieve the therapeutic goal: activity for positive emotion, social activity, cognitive activity, and physical activity. Activities aimed at positive emotion included touching the dog, singing a song, massaging the dog, playing with the dog (ball, loop, game), and artistic creation (dot art). Social activity involved introducing, greeting, praising, thanking, helping, talking, making appropriate physical and eye contact, and cooperating in the games with each other and the dog. Cognitive activity included questions and answers, training the dog, orienting the content of activity, playing a cognitive game (puzzle, triangle, and memory card), and writing a worksheet. Physical activity involved walking, handling, feeding, grooming, dressing, and doing exercises with the dog. Each activity was performed for three sessions with gradually increasing levels of difficulty.

Therapists gave feedback on what the group did during the therapeutic activities, asked how they felt with the dog, and previewed the content of the next session.

### Data Analysis

Statistical analyses were conducted using Statistical Package for Social Sciences (SPSS, version 25.0; Chicago, IL, USA). Data from groups A and B of the AAT condition were analyzed collectively. Categorical variables (e.g., sex and level of education) were converted to percentages and compared using the chi-square test. Given the small sample size, the Shapiro–Wilk test was used to test the normality of the data. As the data fit a non-normal distribution, we used the non-parametric statistics. The continuous variables of the outcome measures (PANSS, DASS, and CHI scores) are presented as the median and interquartile range (IQR) and compared with the Mann-Whitney *U*-test. All tests were two-tailed with a probability ≤ 0.05 (p ≤ 0.05) considered reflective of significance. Additionally, the effect size of success rate difference (SRD) (e.g., treatment group success rate–control group success rate) were calculated for all significant findings (44), with values of 0.11–0.27, 0.28–0.43, and >0.43 categorized as small, moderate, and large effect size, respectively (45).

### Ethics

The study was approved by the local ethics committee of the Chang Gung Medical Foundation Institutional Review Board (No. 202000549B0C601). All participants provided written informed consent. The study was registered at ClinicalTrials.gov (Identifier: NCT04476836).

## Results

### Sample Characteristics

A final sample of 40 participants (20 per group) was analyzed (Figure 1). No participants dropped out. There were no significant differences between the groups at baseline regarding age, sex, language, level of education, marital status, and living condition (Table 1). At pre-test, there were no significant differences between the AAT and control groups in the PANSS scores (*p* = 0.340), DASS (*p* = 0.659), and CHI (*p* = 0.659).

**Table 1:** Sample characteristics.

	**AAT (*n* = 20) f (%)**	**TAU (*n* = 20) f (%)**	***P* value**
Gender			0.057
Male	6	12	
Female	14	8	
Age median (IQR)	55.3 (16.0)	54.1 (18.5)	0.445
40–49	7	7	
50–59	5	8	
60–71	8	5	
Language			0.633
Chinese	17	18	
Taiwanese	3	2	
Education			0.690
Uneducated	1	0	
Elementary school	3	3	
Junior high school	3	6	
Senior high school	9	7	
University/college	4	4	
Condition of marriage			0.327
Single	10	14	
Married	5	2	
Divorced	5	3	
Widowed	0	1	
Condition of living			0.598
live alone	1	1	
not alone	18	19	
Other	1	0	
Defined Daily Dose	1.1 (1.0)	1.3 (0.8)	0.289

### PANSS Scores

The change in the total PANSS score of the AAT group revealed a significant improvement compared to control group (*p* = 0.001). Moreover, the positive, negative, and general psychopathology subscale revealed a significant between-group difference (*p* < 0.001). The AAT group had less psychotic symptoms after the intervention than the control group. The change score in negative subscale presented a large effect size (SRD = 0.5), and the change score for total score, positive subscale, and general psychopathology subscale revealed a small effect size (SRD = 0.15–0.25).

### DASS Scores

The change scores of stress subscale in DASS revealed a significant between-group difference (*p* = 0.012), subjects in AAT group had less stress after the intervention compared to the control group. The change score for stress subscale revealed a small effect size (SRD = 0.15). Moreover, a decreasing trend in anxiety and depression were observed in the AAT group.

### CHI Scores

There were no statistically significant differences in the change score of CHI between the AAT and control groups (*p* = 0.461). However, there was an increasing trend of change scores in the AAT group, with higher well-being in the posttest (see Table 2).

**Table 2:** Median and interquartile range of PANSS, DASS, and CHI change scores between pre-test and post-test for the AAT group and control group.

		**AAT group (** ***n*** **=** **20)**		**Control group (** ***n*** **=** **20)**		***P* value**	**SRD**
PANSS		Median	IQR	Median	IQR		
total score	Pre	11.5	56.8–79.0	11.5	56.3–66.8	0.314	
	Post	11.0	50.5–69.8	11.5	53.8–72.5	0.925	
	Change	−1.0	−12.5–−3.0	0	−1.0–4.8	0.001	0.25
positive subscale	Pre	19.0	9.0–20.3	20.5	8.0–13.8	0.565	
	Post	16.5	8.3–17.0	20.5	8.0–14.8	0.211	
	Change	−3.0	−2.0–0	0	0–1.0	<0.001	0.15
negative subscale	Pre	33.0	16.3–26.8	30.0	16.0–23.8	0.211	
	Post	29.5	14.0–21.5	29.5	14.3–24.0	0.461	
	Change	−3.0	−5.0–−1.3	0	−0.8–0.8	<0.001	0.50
general psychopathology subscale	Pre	66.5	28.3–40.3	60.5	27.0–34.0	0.211	
	Post	57.5	23.3–33.8	60.0	27.0–33.0	0.383	
	Change	−7	−6.3–−1.0	0	−0.8–2.0	<0.001	0.20
DASSstress	Pre	6.0	2.3–11.3	5.5	1.3–10.8	0.659	
	Post	7.0	0.3–9.8	7.5	5.0–12.8	0.341	
	Change	−1.0	−2.0–1.0	1.5	−0.8–4.0	0.012	0.15
DASS	Pre	4.5	2.3–7.0	5.0	0.3–7.8	0.904	
anxiety	Post	4.5	2.0–7.0	5.5	2.3–10.0	0.512	
	Change	0	−1.8–1.0	1.0	−1.0–1.8	0.289	
DASS	Pre	5.0	2.3–8.8	4.5	1.0–7.0	0.620	
depression	Post	6.0	0.5–7.0	7.0	2.0–11.5	0.289	
	Change	0	−0.8–1.8	0.5	−0.8–5.0	0.265	
DASS	Pre	15.0	6.8–26.8	15.0	3.3–24.0	0.659	
total	Post	18.0	2.8–27.0	22.5	9.8–30.5	0.301	
	Change	−0.5	−3.8–3.3	2.5	−2.0–10.8	0.114	
CHI	Pre	42.0	35.8–50.0	41.0	32.8–54.5	0.659	
	Post	43.0	35.8–64.3	41.0	34.0–55.8	0.512	
	Change	1.5	−0.8–8.8	0	−3.5–6.5	0.461	

## Discussion

Compared to the control group, the AAT group showed more significant improvements in negative symptoms, with a large effect size between the two groups. A previous study reported that participants given AAT showed a greater improvement in hedonic tone than the controls, with no significant effect on avolition. However, there was a trend toward improvement in avolition (25). Other studies demonstrated that participants received AAT showed significant improvements in negative symptoms after the intervention, but there were no significant differences than control groups (20, 23). It might be possibly due to the small sample size in previous studies (21 and 24, respectively) (20, 23). Besides, no study assessed the effect size between groups, and only one study has found a large effect size within the AAT group (20). Our study recruited relatively larger sample size, and the results revealed that AAT may improve the negative symptom of patients with schizophrenia.

Improvements in negative symptoms for the AAT group may be underpinned by three core mechanisms. First, therapy dogs acted as social catalysts or mediators to increase social interactions with therapist and patients (20, 46). Therapy dogs have been shown to increase verbal interactions (47), initiation, and participation in longer conversations in the older adults (48). Therefore, AAT improved negative symptoms including poor rapport, lack of spontaneity, and flow of conversation. Second, therapy dogs provided companionship and emotional support in the context of the AAT activities (49), leading to improvements in the apathetic social and emotional withdrawal, and blunted affect. Third, animals are known to help people release oxytocin (31, 32, 50), thereby reducing negative symptoms for patients with schizophrenia (51, 52). However, current evidence remains insufficient and these mechanisms warrant further investigation.

The other core finding was that of the significant effects on positive symptoms and general psychopathology with small effect size between the two groups. A previous study has revealed similar results and supports our finding (53). However, another study presented different results, wherein AAT showed no significant improvements in positive symptom and general psychopathology compared to controls (20, 23). As such, compared to previous results (20, 23), in our study, the AAT group improved more substantially in positive symptoms and general psychopathology. This may be due to our therapist ensured that participants felt well-oriented and that the sessions were realistic during the AAT sessions, clarifying participant conversations. This was not clearly mentioned in previous work. Thus, our study showed that AAT has more significant effects on positive symptoms (such as hallucinations, and conceptual disorganization). In addition, our activities included exercise in AAT, possibly helping to improve general psychiatric symptoms (such as tension, posturing, motor retardation, and impulse control).

Furthermore, as number of samples in past studies was small (20, 23), it remains difficult to determine the effects on positive and general psychopathology symptoms. The benefit of AAT on general positive symptoms and the psychopathology of schizophrenia is worth further investigating. Finally, our study provides initial evidence that AAT may slightly improve positive and general psychopathology symptoms of patients with schizophrenia.

Our findings revealed a significant decrease of stress in the AAT group than in the control group, alongside a small effect size between the two groups. A previous study demonstrated that their AAT group revealed a significant decrease in cortisol, considered as a decrease in stress, but mentioned no comparison between groups (20). Other AAT studies demonstrated stress reduction in patients with post-traumatic stress disorder (54, 55) and dementia (56). Reduced stress-related hormonal responses in patients after AAT may be explained by three core mechanisms. First, the relaxing human–animal bond acted via the adrenal gland and other corticosteroids, the release of oxytocin, dopamine, and endorphins, which may reduce arterial pressure and cardiorespiratory rates, thus leading to decrease stress (20, 29). Second, our AAT session incorporated singing, massaging, artistic creation, and exercises which may help patients reduce stress, as confirmed by previous studies (57–60). As such, our study provided initial evidence that AAT may slightly decrease stress in patients with schizophrenia.

Our study revealed no significant difference between the two groups, although there was a trend toward decreased anxiety and depression in the AAT group. Some previous studies demonstrated similar results. In these studies, reduced anxiety was twice as great (34) and there was a significant decrease in depression in patients with schizophrenia after AAT (29), but no significant difference between the two groups (29, 34), consistent with our finding. However, another study found that neither the AAT nor the control group showed significant improvements in anxiety and depression (20). This study provided limited information regarding anxiety and depression scores because some patients could not fully understand all items' meanings (20). Therefore, it is difficult to compare the difference in results to ours.

Our study revealed that AAT had limited effectiveness in anxiety and depression, possibly because the pre-test levels of depression and anxiety subscales in our participants were mild to normal (61). As such, they had less depression and anxiety, meaning that the AAT had limited effectiveness. In the future, schizophrenic patients with moderate to extremely severe depression and anxiety can be recruited to further assess the effectiveness of AAT on depression and anxiety.

An increasing trend in well-being could be observed for the AAT group, but there was no significant difference between the two groups. Our study is the first to present the effects on well-being among patients with schizophrenia. A previous study showed that participants with mental illness reported the experience as enjoyable and interesting at the end of the AAT (29), partially supporting our findings. A few studies showed indirect evidence that participants with schizophrenia and dementia experienced a better quality of life after the AAT (19, 20, 62), which may further improve their sense of well-being (63). One reason for the improvement in their well-being may be that in company of dogs, participants experienced more love and support (64, 65), sharing feeling with the dogs. However, the effect and mechanism of AAT on well-being warrant further investigation.

There are three strengths to our study. First, our study used the trans-disciplinary approach enlisting both of animal-assisted therapist and occupational therapist, based on their unique knowledge and skills, together determine the therapy that would most benefit patients. Second, the AAT activity was structured and diversified, including activity for positive emotions, social activity, cognitive activity, and physical activity to achieve therapeutic goals. Lastly, there had been few controlled studies of AAT in the community other than in a hospital setting, and in our study no participants dropped out, thereby ensuring the feasibility and high adherence to the AAT for hospitalized psychiatric patients.

There are also a number of limitations that should be mentioned. Owing to the nature of the intervention, it was not possible to blind the participants and therapist to the allocations. Moreover, our study presented only short-term effects; and future research could extend the time of follow-up to ensure the long-term effects of AAT. Finally, we suggest using larger sample sizes and collecting biomarkers in further studies to minimize research bias.

## Conclusion

Animal assisted therapy can be effective at reducing of psychopathology symptoms and stress in middle-age and older adults with schizophrenia, particularly improving negative symptoms. In addition, there may also be improvements in anxiety, depress and well-being. Therefore, AAT represents a potential adjunct therapy for patients with schizophrenia. However, future higher quality research is required in order to better understand the mechanisms underpinning AAT and studies assessing biomarkers are needed.

## Data Availability Statement

The raw data supporting the conclusions of this article will be made available by the authors, without undue reservation.

## Ethics Statement

The studies involving human participants were reviewed and approved by Chang Gung Medical Foundation Institutional Review Board. The patients/participants provided their written informed consent to participate in this study.

## Author Contributions

T-TC, C-FH, and C-RC conceived, designed, and conducted this study. T-TC, M-LC, and C-RC conducted intervention. W-TT and C-FH contributed in the statistical analysis and interpretation. T-TC, T-LH, and C-RC drafted the manuscript. All authors approved this manuscript.

## Conflict of Interest

The authors declare that the research was conducted in the absence of any commercial or financial relationships that could be construed as a potential conflict of interest.

## Publisher's Note

All claims expressed in this article are solely those of the authors and do not necessarily represent those of their affiliated organizations, or those of the publisher, the editors and the reviewers. Any product that may be evaluated in this article, or claim that may be made by its manufacturer, is not guaranteed or endorsed by the publisher.
